# Technical Approaches to Predicting Acute Deterioration in Pediatric Inpatients: Protocol for a Scoping Review

**DOI:** 10.2196/100097

**Published:** 2026-09-24

**Authors:** Sophia Lau, Sophie Manami Orgler, Samiran Ray, Alexander Philip Yehuda Brown

**Affiliations:** 1 Medical School, Faculty of Medical Sciences, University College London, London, England, United Kingdom; 2 Paediatric Intensive Care Unit, Great Ormond Street Hospital, London, England, United Kingdom; 3 Institute of Child Health, University College London, 30 Guilford St, London, England, WC1N 1EH, United Kingdom, 44 7747020589

**Keywords:** pediatrics, machine learning, early warning score, patient acuity, clinical deterioration, digital health, algorithms

## Abstract

**Background:**

Early warning systems are widely used to detect acute clinical deterioration, which may be defined as a significant worsening in health over a few hours that may lead to adverse outcomes such as code blue activation, unplanned intensive care unit admission, or death. These systems rely on the regular measurement of physiological parameters, such as heart rate and blood pressure, which are converted into warning scores using deterioration prediction algorithms (DPAs). A range of DPAs are currently in use, most commonly simple track-and-trigger tools or summative scoring systems. More complex machine learning approaches have been proposed that may improve prediction accuracy. However, heterogeneity in outcome definitions and reported model performance metrics hinders the evidence synthesis needed to support the deployment of proposed models in clinical contexts.

**Objective:**

This scoping review aims to identify the range of DPAs developed for use in pediatric inpatient early warning systems, as well as operational definitions of deterioration and reported performance metrics.

**Methods:**

The review will follow the Joanna Briggs Institute methodology for scoping reviews and the PRISMA-ScR (Preferred Reporting Items for Systematic Reviews and Meta-Analyses extension for Scoping Reviews) reporting guidelines. The population of interest is hospitalized children. The concept under review is DPAs, defined as decision-support tools that use routinely monitored physiological parameters to alert clinicians to worsening clinical status. The context will be inpatient ward settings, excluding emergency departments, neonatal units, and intensive care environments. Studies will be identified from searches of the MEDLINE, Embase, HMIC, Scopus, Web of Science, Cochrane, and ACM DL databases. Studies will be screened by 2 independent reviewers against the inclusion and exclusion criteria. A broad range of study types, including prospective and retrospective analyses, will be eligible for inclusion. Data on the choice of algorithmic approach, definition of deterioration, and reported performance metrics will be collated and presented descriptively in tabular and narrative formats.

**Results:**

At the time of submission, the protocol has been registered and the search strategy finalized. A formal database search was carried out in May 2026. Screening and data extraction are expected to be completed by winter 2026, after which the findings will be published.

**Conclusions:**

This protocol describes the planned scoping review of DPAs for pediatric inpatient care. The completed review will summarize the types of algorithms evaluated, the outcomes used to define deterioration, and the performance metrics reported. These findings will support further evidence synthesis in this emerging field.

## Introduction

Early warning systems are widely used across health care to support the early detection of deterioration [1] for patients in hospital. Typically, these systems comprise 3 elements: regular measurement and recording of physiological parameters such as heart rate, blood pressure, and blood oxygen saturation; calculation of a risk score using a deterioration prediction algorithm (DPA); and subsequent escalation of care by clinicians in response to raised risk scores, often in accordance with predefined rules. The early warning scores form part of a wider system of rapid response [2], which may include parallel systems of escalation initiated by a member of the care team, the patient, or their relative [3]—independent of the risk score. Once a concern is raised by the “afferent” limb of this system [4], interventions are carried out by clinicians either from the parent medical team or a dedicated critical care outreach team [5,6].

Pediatric early warning systems (PEWS), developed in the early 2000s [7,8], were designed for paper charts and thus use simple DPAs. Track-and-trigger systems define thresholds at which individual parameters should activate an alert (such as tachycardia above a certain rate). Summative systems coarsely categorize each parameter into a subscore; these subscores are then summed to give an overall early warning score. Many recent PEWS incorporate both a rules-based and a summative element in their DPAs.

PEWS were initially developed locally, producing considerable variation in score structure, thresholds, chart design, and escalation pathways across hospitals [8-11]. In response to this heterogeneity, recent national initiatives—most notably the National Paediatric Early Warning System (National PEWS) in the United Kingdom [12]—have sought to create a standardized approach. National PEWS emphasizes a safety-focused approach that prioritizes operational reliability, reduction in unwarranted variation, human factors, and the practical realities of bedside care. While standardization addresses implementation challenges, it does not examine whether early warning systems can be improved using alternatives to the simple DPAs at the heart of the system.

As patient record systems move toward universal computerization in industrialized countries, more complex DPAs may be implemented to improve the accuracy of early warning systems. AI-based and machine learning (ML)–based deterioration prediction models, automated monitoring platforms, and digital integration tools have been proposed to identify patients at risk more accurately than traditional score-based DPAs in a variety of contexts [13]. These models often draw on large datasets and advanced predictive analytics to identify risk earlier and more accurately [13,14]. Despite individual reports of improved performance metrics, there have been no systematic reviews addressing the optimal choice of DPAs, or indeed if any of these reliably outperform simple rule-based or summative approaches for predicting acute deterioration in hospitalized children.

Reporting guidelines for AI or ML in health care such as TRIPOD-AI (Transparent Reporting of a Multivariable Prediction Model for Individual Prognosis Or Diagnosis With Artificial Intelligence) [15] are still relatively new; studies developing or evaluating predictive models have therefore reported outcomes inconsistently and lack uniform adherence to best-practice standards [16]. This inconsistency is exacerbated by the range of methodological approaches that have been taken including rule-based scores, classical ML, and deep learning, whose reporting practices and evaluation standards differ widely. Finally, there are a variety of event types used to operationalize the concept of “deterioration” [10], including code blue calls, cardiac arrest, unplanned transfer to intensive care, emergency interventions, or death. This further complicates any attempt to directly compare the effectiveness of DPAs.

Guided by the Joanna Briggs Institute (JBI) methodological framework [17,18] and PRISMA-ScR (Preferred Reporting Items for Systematic Reviews and Meta-Analyses extension for Scoping Reviews) guidance [19], this scoping review will map the range of techniques and evaluation approaches used in existing research on DPAs for pediatric inpatients. We will address the following questions:

- Which kinds of algorithms have been used as DPAs?
- Which definitions of deterioration have been used in these studies?
- Which metrics have been reported in assessment of DPA performance?

We will also describe how DPA choice co-occurs with reported performance and calibration metrics and definitions of deterioration through descriptive mapping and cross-tabulation. Our review will characterize the methodological landscape of DPAs for pediatric inpatients, including how algorithms are evaluated and deterioration is defined. This will inform future evidence synthesis aimed at evaluating the comparative performance of different deterioration prediction approaches.

Prior to the initiation of this review, a preliminary search of MEDLINE, the Cochrane Database of Systematic Reviews, and *JBI Evidence Synthesis* was conducted to identify existing evidence synthesis in this area. Several reviews were identified focusing on PEWS; however, none were identified that specifically addressed the effect of DPA choice on predictive accuracy. One study was identified that addresses questions similar to our own in the field of neonatology [20], and a protocol was identified that focused solely on adult patients [21]. Both of these studies exclude pediatric inpatients, the population of interest in this proposed review.

## Methods

### Overview

This scoping review will be conducted in accordance with the JBI methodological framework [18]. The PRISMA-ScR checklist [19] will be used to ensure the completeness of reporting and transparency. A completed checklist is included in Checklist 1. A cloud-based tool (Rayyan, 16) will be used to manage deduplication, screening, and data extraction.

### Protocol Registration

This scoping review was preregistered with the Open Science Framework (OSF) Registry [22]. Preregistration ensures openness and reproducibility by providing a predefined description of the review objectives, methods, and planned analyses. This record also allows external evaluation and supports future updates or amendments if required.

### Eligibility Criteria

Study eligibility will be defined using the population, concept, and context format, as recommended by the JBI guideline. This is summarized in Table 1 and expanded further in the sections below.

**Table 1.** Population, concept, and context (PCC) summary.

Domain	Definition
Population	Hospitalized children
Concept	Algorithms used to predict acute clinical deterioration
Context	Patients admitted to pediatric wards or units

#### Population

The population of interest is hospitalized children, encompassing all pediatric inpatients regardless of underlying diagnosis, age group, or clinical specialty. Due to international and regional variation in practice when defining the boundary between childhood and adulthood, we will include any study that self-defines as pediatric, as opposed to imposing a particular age cutoff.

Studies involving mixed-age populations will be included if data for children are reported as a separate subgroup analysis.

#### Concept

The concept of this study is technical approaches used by DPAs. DPAs will be broadly defined as systems where physiological variables are judged by the reviewers to constitute a substantial component of the predictor set used to estimate the risk of acute clinical deterioration. Additional variables, such as laboratory tests, comorbidities, medications, imaging, or administrative data, may also be included alongside physiological data. Models in which physiological data play only a minor role (eg, a single measurement of weight used to contextualize laboratory test results), or are absent entirely, will be excluded. These systems may operate via simple rules-based approaches, sometimes referred to as “track-and-trigger” [8]; traditional statistical approaches such as logistic regression or ML tools using a variety of approaches suitable for tabular data, such as gradient-boosted trees, neural networks, or support vector machines.

#### Context

The context includes inpatient hospital settings to which patients are formally admitted, such as general pediatric wards, inpatient assessment or observation units, and high-dependency units. Emergency departments or primary care settings, where undifferentiated patients first present for assessment, will be excluded as the focus of this review is on deterioration prediction during inpatient care rather than initial assessment, triage, or disposition. Intensive care units (defined by the availability of invasive physiological support such as endotracheal intubation or vasoactive medications) will also be excluded, as patients in these settings typically receive intensive physiological monitoring and organ support, making deterioration prediction a fundamentally different task from that in general inpatient care. Studies in mixed settings will only be eligible where results for the eligible inpatient settings are reported separately.

#### Types of Studies

This review will consider a broad range of study designs, including randomized controlled trials, nonrandomized controlled trials, before-and-after studies, and interrupted time-series studies that evaluate DPAs. Observational studies, such as cohort, case-control, and cross-sectional designs, will also be eligible, as will model development, validation, and implementation. Both prospective and retrospective studies will be included.

Studies conducted using simulated or synthetic data will be considered. The use of synthetic datasets is becoming increasingly common in the development and evaluation of health care AI systems (eg, [23]) with growing support from regulators, particularly during early-stage development [24]. As the objective of this scoping review is to characterize the landscape of DPAs, along with chosen outcomes and reported metrics, rather than to compare model performance, we consider this work to be in scope.

Qualitative and mixed-methods studies will be considered where they meet the population, concept, and context criteria defined above. Systematic reviews will not be included but may be used for citation searching.

Studies conducted in any geographical region will be eligible, provided that the study is published in English and available in full text. We will restrict our search to papers published on or after January 1, 2000, up until the date of the final search, anticipated to occur in winter 2026.

### Exclusion Criteria

Studies will be excluded if they focus exclusively on neonatal or adult populations, or are based in emergency or intensive care units, or community settings. Mixed population studies may be considered where pediatric inpatient results are reported separately or as a subgroup analysis.

Studies describing prognostic scores related to specific diseases or modes of presentation will not be considered (such as risk of mortality in a particular form of leukemia), as the focus of this review is on early warning of acute deterioration for a general population, rather than disease-specific prognostication. Studies that consider a disease-enriched population where the outcome is general deterioration (such as studies examining the performance of a PEWS system in a pediatric inpatient oncology ward) will, however, be considered.

We will exclude studies that do not report any algorithmic performance metrics or clinical outcomes relating to acute deterioration, such as those describing unrelated interventions; general monitoring without predictive intent; implementation studies that do not evaluate algorithmic performance or clinical deterioration outcomes; human factors, workflow, or usability studies; or nonclinical tools.

We will exclude editorials, letters, case reports, commentaries, narrative reviews, conference abstracts, as well as studies not published in English and studies where the full text is unavailable.

Gray literature will be excluded to maintain consistency in methodological reporting and to ensure the inclusion of only studies with sufficient detail and peer-reviewed quality standards, particularly given the volume of brief, partial, non–peer-reviewed outputs in this field (including online repositories, technical reports, tutorials, and data science competition entries). While this approach may underrepresent some recently proposed AI and ML approaches, many gray literature reports provide insufficient methodological detail for consistent extraction of algorithm characteristics, outcome definitions, validation strategies, and model evaluation metrics. Their inclusion could therefore introduce systematic differences in the completeness of extracted data and reduce the comparability of studies included in the review.

Where multiple studies are identified using the same underlying dataset or patient cohort (eg, publications arising from data-science competitions using open-source datasets), these will be assessed for material novelty before inclusion. Studies sharing datasets will be considered to make a materially novel contribution if they (1) pose a different research question; (2) use a different operational definition of deterioration or prediction horizon; (3) implement a different modeling approach; (4) implement nontrivial changes to imputation or data preprocessing strategies; (5) provide novel or independent validation; or (6) provide another clearly identifiable novel methodological or clinically relevant finding, including replication studies. Studies that do not meet any of these criteria will be excluded from the primary report. Excluded studies will be listed alongside the rationale(s) for exclusion.

Inclusion and exclusion criteria are summarized in Textbox 1.

Textbox 1. **Summary of inclusion and exclusion criteria.**

**Inclusion**

- Hospitalized pediatric inpatients
- Mixed studies where pediatric data are reported separately
- General deterioration prediction algorithms where physiological variables constitute a substantial component of the predictor set
- Models that incorporate additional predictors such as laboratory tests, medications, comorbidities, imaging, or administrative data, alongside physiological measurements
- Inpatient ward settings, including ambulatory care or assessment units
- Mixed studies where inpatient data are reported separately
- Randomized, nonrandomized, observational, model development, validation, and implementation studies
- Prospective, retrospective, simulated, or synthetic data studies
- Qualitative or mixed-methods studies meeting population, concept, and context criteria
- Reports at least 1 algorithm performance metric
- Full-text English-language peer-reviewed studies published on or after January 1, 2000

**Exclusion**

- Neonatal-only or adult-only studies
- Mixed studies in which pediatric data are not reported separately
- Disease-specific prognostic models
- Models where physiological variables form only a minor component of the predictor set, or are absent entirely

- Emergency department, intensive care unit, community settings
- Mixed studies in which inpatient data are not reported separately
- Editorials, letters, case reports, commentaries, narrative reviews, conference abstracts, gray literature
- Studies sharing underlying datasets which do not make materially novel contributions in methodology
- No algorithmic performance evaluation
- Non-English, full text unavailable, published prior to 2000

### Data Sources and Search Strategy

Guided by the JBI framework, a search strategy was developed to identify published studies meeting the inclusion and exclusion criteria defined above.

First, key papers were identified by discussion among the authors. Preliminary search strategies were developed for MEDLINE (Ovid), Embase (Ovid), and HMIC (Ovid) and iterated to ensure that all identified studies of interest were included.

This resulted in a proposed search strategy, which was then tested for the inclusion of further papers of interest identified by colleagues, resulting in the final search strategy, which additionally includes searches for Cochrane, Scopus, ACM Digital Library, and Web of Science (Clarivate; Multimedia Appendix 1). Search terms include a mix of controlled vocabulary (MeSH/Emtree terms where possible), as well as free-text keywords, and are applied to the title and abstract of articles in the respective databases. The search strategy was designed to prioritize sensitivity, given the heterogeneity of terminology used in this field. The strategy was tested for feasibility; 6744 articles were identified after automated deduplication.

Finally, a secondary search of references will be conducted, using any studies identified for inclusion.

The literature search will not be rerun prior to the completion of the review. The review will synthesize evidence identified within the predefined search period, providing a reproducible snapshot of the methodological landscape that may inform future evidence syntheses.

### Screening and Selection Procedures of Eligible Studies

Following the search, all identified citations will be collated and uploaded into Rayyan [25], and duplicates will be removed. A pilot screening phase will then be conducted to ensure clarity and consistency in applying the eligibility criteria. During this phase, 2 independent reviewers will screen at least 25 randomly selected titles and abstracts, including at least 3 studies included at this stage by one or more reviewers. A minimum consensus threshold of 75% agreement will be required to proceed to formal screening, as recommended in the scoping review protocol section of the JBI manual for evidence synthesis [17]. If this threshold is not achieved, the inclusion and exclusion criteria will be discussed, clarified, and refined as necessary before repeating the pilot screening phase.

Following successful piloting, titles and abstracts will be screened independently by 2 reviewers against the predefined inclusion and exclusion criteria for potential inclusion. The full text of studies identified at the screening stage will then be assessed independently. Reasons for exclusion of studies at the full text assessment stage will be recorded and reported in the final scoping review.

Any disagreements that arise at the initial or full-text screening stages of the selection process will be resolved through discussion or, where necessary, consultation with an additional reviewer. The results of the search and the study inclusion selection process will be reported in full in the final scoping review and presented using a PRISMA-ScR flow diagram.

### Data Extraction

Data extraction will be performed within Rayyan, aligned with JBI guidelines. The fields to be extracted are presented in detail in Table 2. Two reviewers will independently record data including:

- Basic study characteristics (eg, author, year, country, etc).
- Population and context, including whether the study was conducted on a mixed population where a pediatric inpatient population was identified as a distinct subgroup (mixed studies without this identification will be excluded entirely).
- Concept: The technical approach or approaches used as DPAs in the study, classified according to a unified taxonomy (Table 3).
- Clinical outcome or outcomes: The operational definition of deterioration in the study, such as unplanned intensive care admission, mortality, in-hospital emergency call, or escalation of care. This will be classified into a multilabel framework comprising commonly used outcome types, while also being recorded verbatim.
- Number of outcome events, as well as population size denominator (patients, admissions, and/or observations). Event rates (per patient, admission, or observation) will be recorded where provided or calculated if the data permit unambiguous calculation. Model performance metrics such as the area under the receiver operating characteristic curve, area under the precision–recall curve (AUPRC), sensitivity, specificity, *F*_1_-score, positive predictive value (PPV), or other metrics.
- Handling of missing data, including whether complete-case analysis, native handling, single imputation, multiple imputation, model-based imputation, informative missingness indicators, or other approaches were used, or whether missing data handling was not described.
- Calibration metrics, including whether a calibration curve was reported and any reported calibration measures such as calibration slope, calibration intercept, Brier score, expected calibration error, or other calibration metrics.
- Model validation approach, including whether validation was internal or external. For internally validated models, the validation strategy will be extracted (eg, random split, temporal split, k-fold cross-validation, nested cross-validation, bootstrapping, repeated holdout validation, or other approaches). Where applicable, we will also record whether model performance was evaluated using an independent test set distinct from the data used for model development (including hyperparameter selection).

As this is a scoping review, no formal critical appraisal will be conducted, consistent with JBI guidance.

**Table 2.** Data extraction fields^a^.

Section and details	Notes
Basic study characteristics	
Title	
Authors	
Citation reference (DOI)	
Year of publication	
Country or countries	In which research was conducted
Study design	Prospective, retrospective cohort, case-control, or other
Scope	Single-center, multicenter
Study center type	Secondary, tertiary, mixed academic, or nonacademic center
Simulated or synthetic dataset used?	Yes, if data are even partially synthetic. No, if real-world data exclusively
Population	
Pediatric population	However defined
Neonatal or postnatal population	Studies will be excluded if they only include these contexts, or if an inpatient context is not reported as a separable subgroup
Adult population	Studies will be excluded if they only include these contexts, or if an inpatient context is not reported as a separable subgroup
Definition of “pediatric”	How is the pediatric population defined—age cutoff or other method. Free text recording if not age cutoff
Maximum age	Distribution measures of age data
Minimum age	Distribution measures of age data
Median age	Distribution measures of age data
Age IQR	Distribution measures of age data
Context	
Includes emergency department?	Studies will be excluded if they only include these contexts, or if an inpatient context is not reported as a separable subgroup
Includes intensive care?	
Setting	Typically, inpatient wards; may be other contexts such as short stay clinical decision units; ambulatory care. Outpatient or home-hospital will be excluded altogether
Concept*	
DPA^b^ model type or types	As specified in the report. Model type will be recoded verbatim, as well as classified according to a unified taxonomy (Table 2)
Clinical outcome definition	
Verbatim outcome definition	Complete wording of the deterioration outcome as reported in the study
Clinical outcome definition (multiselect)	
Unplanned admission to PICU^c^	Or other intensive care unit, for example, local adult intensive care
Mortality	
In-hospital emergency call	Code Blue, 2222 call
Escalation of care (specify)	For example, increased respiratory support
Other (specify)	
Outcome	
Number of outcomes observed	Recorded if reported
Number of patients	Recorded if reported
Number of admissions	Recorded if reported
Number of observations	Recorded if reported
Outcome rate (per patient)	Event rates will be reported if provided, or calculated where the reported data permit unambiguous calculation
Outcome rate (per admission)	Event rates will be reported if provided, or calculated where the reported data permit unambiguous calculation
Outcome rate (per observation)	Event rates will be reported if provided, or calculated where the reported data permit unambiguous calculation
Prediction horizon	Time-to-event window (in hours)
Model performance metrics*	
Sensitivity, recall, or true positive rate (TPR)	
False negative rate (1-TPR)	
Specificity or true negative rate (TNR)	
False positive rate (1-TNR)	
Positive predictive value (PPV) or precision	
False discovery rate (1-PPV)	
Negative predictive value (NPV)	
False omission rate (1-NPV)	
*F*_1_-score	
AUROC^d^	
AUPRC^e^	
Likelihood ratio	
Odds ratio	
Other (specify)	
Imputation*	
Complete-case analysis	No imputation
Native handling	Model does not require imputation
Single imputation	
Multiple imputation	
Model-based imputation	
Missingness indicator or informative missingness	
Missing data not described	
Other (specify)	
Calibration*	
Calibration curve present in paper	
Calibration slope	
Calibration intercept	
Brier Score	
Expected calibration error	
Other calibration metric (specify)	
Validation type*	
Internal	Model evaluated using data from the same source as the development dataset
External	Model evaluated using data from another institution, site, or department
Validation test set*	
Independent test set	Was a distinct dataset used to characterize model performance, and not for any model development (including hyperparameter selection)
Internal validation approach*	
Temporal	Train or test split based on time
Random	Random train or test split
Stratified	Random split preserving outcome prevalence or other characteristics
K-fold cross-validation	Includes repeated application across k folds
Nested cross-validation	Inner loop for model selection or hyperparameter tuning and outer loop for performance estimation.
Bootstrapping	Internal validation using bootstrap resampling
Repeated holdout validation	Also known as Monte Carlo cross-validation
Other validation strategy (specify)	

a Note that for clinical outcomes and model performance metrics, the focus of this review will be on which items were reported, rather than the numerical values of these data. Equivalent terms have been grouped together in model performance metrics. Model-level data fields are denoted with an asterisk (*).

b DPA: deterioration prediction algorithm.

c PICU: pediatric intensive care unit.

d AUROC: area under the receiver operating characteristic curve.

e AUPRC: area under the precision–recall curve.

**Table 3.** Proposed taxonomy of deterioration prediction algorithms (DPAs), based upon [26]^a^.

Model family	Examples
Rules based	Track and trigger (no summative scoring) Summative score
Classical statistical	
Trees	Decision tree
Forests	Random forest
Boosted forests	Adaptive boosting Gradient boosted trees Extreme gradient boosting
Other	Logistic regression K-nearest neighbors Naïve Bayes Support vector machine
AI or machine learning	
Neural networks	Fully connected feedforward network or multilayer perceptron (MLP) Transformer
Recurrent neural networks	Recurrent neural network (RNN) Gated recurrent units (GRUs) Long short-term memory (LSTM)
Convolutional neural networks	Convolutional neural network (CNN)
Autoencoders	Variational autoencoder (VAE) Other autoencoder
Generative adversarial networks	Generative adversarial network (GAN)

a This taxonomy may be adapted during the scoping review to reflect additional unanticipated deterioration prediction algorithm methodologies. Categories containing substantial methodological heterogeneity may be subdivided into more specific groups if required.

Studies evaluating multiple DPAs will be included once at the study level. However, data relating to each distinct algorithm—namely model type, imputation strategy, validation strategy, and reported performance metrics—will be extracted separately. Study-level characteristics, including population, setting, study design, outcome definition, and prediction horizon, will be recorded once for each study where a single value is reported. Where studies report multiple values for a study-level characteristic (eg, multiple prediction horizons), all reported values will be extracted and recorded. Any differences in study-level characteristics between models within a study will also be documented.

The draft data extraction form will be pilot tested on a sample of included studies to ensure clarity, consistency, and comprehensiveness. Any discrepancies will be discussed and resolved to refine the tool prior to commencing full data extraction. The extraction form may be iteratively modified during the review process to ensure it captures all relevant information. Modifications will be detailed and reported in the final scoping review. Disagreements that arise during data extraction will be resolved through discussion between the 2 independent reviewers or, if required, with an additional reviewer or reviewers. Where necessary, study authors may be contacted to request missing or additional data.

### Data Synthesis and Reporting

**Figure 1.**
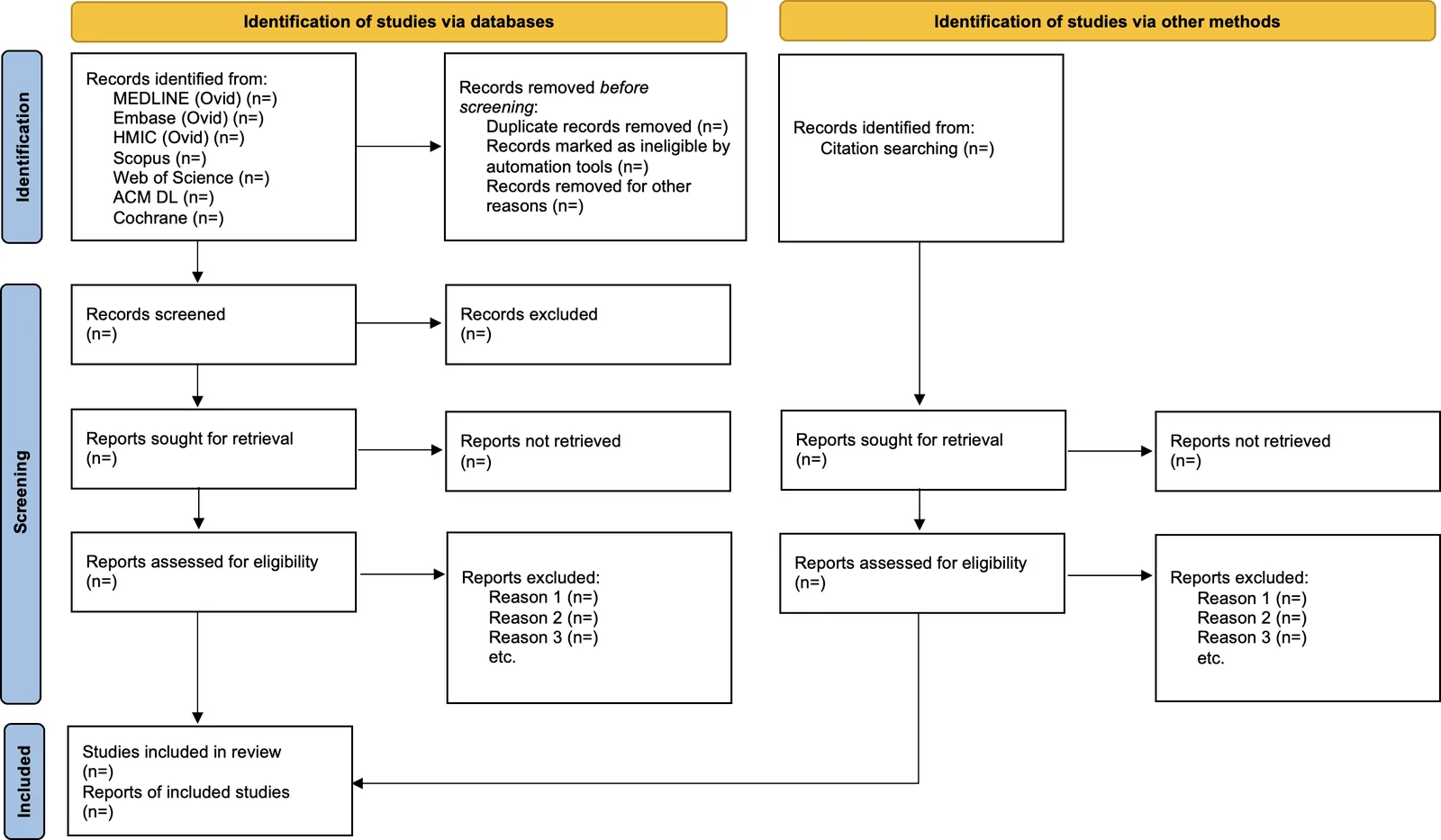
PRISMA (Preferred Reporting Items for Systematic Reviews and Meta-Analyses) flow diagram.

Findings will be reported in accordance with the PRISMA-ScR, including a study selection process presented as a PRISMA flow diagram (Figure 1) and a table of reasons reported for exclusion at the full-text stage.

Characteristics of the included studies will be summarized and presented in tabular form. We will report the number and proportion of studies using wholly or partially synthetic datasets, alongside the number and proportions of studies using exclusively real-world clinical data.

The DPA will initially be extracted verbatim, as reported by the study authors. A taxonomy based on established classes of DPAs [26] will then be used (Table 2) to classify these algorithms into 3 broad groups (rule-based, classical statistical, and AI- or ML-based approaches), and then into more fine-grained subdivisions according to the specific algorithm used.

As the landscape of existing algorithms is not yet known, it is likely that this taxonomy will need to be refined iteratively during data extraction, according to the following rules:

1. Synonymous algorithm names will be merged into existing categories.
2. Previously unrepresented algorithm types may be incorporated as additional categories where required.
3. Categories containing substantial methodological heterogeneity as judged by the authors may be subdivided into more specific groups.

Categories will only be added or removed where this improves methodological coherence. All adjustments and their rationale will be documented in the final review. Subgroup counts will be reported for all levels of the final taxonomy.

The operational definition of deterioration will be recorded using a multilabel extraction framework. Specifically, we will record whether each study defines deterioration to include mortality, unplanned admission to the pediatric intensive care unit, emergency response activation (eg, 2222 or Code Blue), or escalation of care not amounting to pediatric intensive care unit admission [10]. Where escalation of care is included, the specific definition used by the study will also be extracted, as will any other outcome components not encompassed by these categories. This approach permits representation of both single and composite outcome definitions. We will report the proportion of studies including each outcome component (alone or in combination), the proportion using single versus composite outcome definitions, and describe commonly recurring combinations of deterioration outcomes identified across the literature. The complete outcome definition will also be extracted verbatim to preserve study-specific wording, timing requirements, and any hierarchical or composite structure not fully captured by this coding framework.

Event rates will be summarized as reported by the study or calculated, where possible, using the number of events and the denominators used by the original study. We will summarize separately event rates per patient, admission, and observation. Event rates will not be reconstructed from partial, ambiguous, or insufficient information.

Overall proportions will be reported for each model performance and calibration metric, validation type (internal vs external), imputation, and data splitting strategy. Prediction horizons will be presented as a histogram. We will report the proportion of studies that use more than 1 prediction horizon and describe commonly occurring combinations of prediction horizons reported within individual studies.

The co-occurrence of DPA choice and reported performance and calibration metrics, and with operational definitions of deterioration, will be explored using descriptive cross-tabulation. No statistical tests of association or comparative analyses of model performance are planned. The aim is to identify common methodological patterns and potential sources of heterogeneity relevant to future evidence synthesis.

Due to anticipated heterogeneity in modeling approaches and outcome measures, a formal quantitative synthesis aimed at identifying the best performing DPAs will not be undertaken. Instead, the findings will be presented using tables and charts accompanied by a narrative summary highlighting key methodological trends, inconsistencies in reporting, and gaps in the literature relevant to the development of pediatric deterioration prediction systems.

### Dissemination Plan

The findings will be published in a peer-reviewed open-access journal and presented at local and national scientific meetings.

## Results

This protocol was registered on the Open Science Framework in April 2026 [22], following which an initial search was commenced in May 2026, restricted to papers published from 2000 to 2025 inclusive. Prior to publication, an updated search will be conducted to include the most recent literature using the same databases, keywords, and inclusion or exclusion criteria. Newly identified records will undergo the same deduplication, screening, and data extraction steps as the initial search. No further searches will be subsequently conducted.

The review is anticipated to be completed over the course of 6 months, with results finalized for publication by winter 2026. Data will be collated and screened within Rayyan, with results exported in CSV format. Extracted data will be mapped and summarized using tables and charts to present key study characteristics, algorithm types, outcome measures, and performance metrics.

## Discussion

Early warning scores are vital elements of inpatient patient safety systems. Our review will focus on characterizing the range of DPAs used to enhance PEWS, together with the methodological approaches used to develop and evaluate them.

### Principal Findings

We anticipate that this scoping review will demonstrate substantial methodological heterogeneity in the implementation and evaluation of DPAs for hospitalized children. In particular, we expect considerable variation in algorithm types, operational definitions of deterioration, validation strategies, prediction horizons, and reported performance metrics. By systematically mapping these methodological characteristics, the review will identify areas of convergence that may support future quantitative evidence synthesis, as well as aspects of the literature where greater standardization is required.

We anticipate that the large majority of studies included will be retrospective and single-center in design (eg, [8-11]; see also [20,21]). Such methodological patterns, alongside variation in outcome definitions and performance metrics, are likely to limit cross-study comparison and interpretation, as has been found in previous research addressing related questions [10,27]. By cataloging modeling approaches and summarizing how deterioration is defined and performance reported, this review will provide structure to a literature that is currently dispersed and heterogeneous.

Given that these models typically use tabular electronic health record data, we expect many studies to use classical statistical approaches such as logistic regression, as well as more complex ML techniques [20,28]. Core discrimination metrics such as area under the receiver operating characteristic curve and *F*_1_-score will also be valuable for informing future model development and reporting standards. However, while discrimination measures are commonly emphasized, calibration, fairness, and measures of clinical utility may be less consistently reported, limiting the assessment of real-world applicability. Adherence to reporting frameworks such as TRIPOD+AI [15] will help improve the standardization and reporting of these measures in more recent and future research.

The increasing complexity and capacity of AI or ML models suggests a theoretical advantage over traditional early warning systems; many studies may report superior performance for these approaches [14,28-30]. However, the focus of the scoping review will be on providing a structured overview of the methodological landscape, rather than directly quantifying DPA performance. Our work will inform further efforts to define a systemic investigation into whether reported improvements reflect genuine advances in predictive modeling, or differences in study design and outcome definition.

If heterogeneity—in definitions, study designs, and reporting standards—is present to a degree that makes a systematic review infeasible, this would reinforce the need for greater standardization in pediatric deterioration research. Establishing common outcome definitions and core reporting metrics would improve comparability, reproducibility, and ultimately clinical translation that can lead to meaningful improvements in patient safety.

### Limitations

This review has several limitations. Although a comprehensive search strategy will be used, and the review will follow PRISMA-ScR guidance to ensure transparency, some constraints remain. Many included studies are expected to be retrospective and conducted in single centers, which may limit the strength and generalizability of the evidence. The review will also be restricted to English-language publications and studies published from 2000 onwards, which may introduce language bias and exclude earlier relevant work.

This review will exclude gray literature, which may limit the scope of the evidence captured. Many novel ML approaches are reported in non–peer-reviewed contexts, such as technical reports, arXiv submissions, or online repositories. However, these often lack sufficient methodological detail and standardized reporting, which is particularly important in a clinical safety-driven context. Restricting our search to peer-reviewed studies may therefore underrepresent the horizon of emerging techniques but will result in a more consistent and interpretable review that can inform emerging translational efforts.

Additionally, considerable variation is anticipated in modeling approaches, outcome definitions, and reported performance metrics. A formal meta-analysis will not be possible. Instead, our review will be reported narratively, outlining the distributions of these variables. While this will limit direct quantitative comparison between approaches, our findings will inform future evidence synthesis in this area.

### Future Directions

Following the completion of this scoping review, we will use our findings to define a feasible and impactful systematic review aiming to quantitatively compare performance and calibration metrics of DPAs across different model classes.

### Conclusions

Early warning systems play an important role in pediatric inpatient safety; it is natural that approaches that promise to improve the predictive power of such systems may be of great interest in the field. AI and ML have led to the development of increasingly complex models to predict clinical deterioration. However, variation in modeling approaches, outcome definitions, and performance reporting makes comparisons across studies difficult. No ongoing systematic or scoping reviews have been identified that specifically examine these approaches to predict acute deterioration in hospitalized children.

This scoping review will map the range of models used and examine how deterioration outcomes and model performance are defined and reported. In so doing, it aims to clarify the current methodological landscape. We will identify areas of convergence amenable to future quantitative synthesis, as well as gaps in the literature requiring further standardization or primary research.

## Supplementary material

10.2196/100097 Multimedia Appendix 1 Search strategies.

10.2196/100097 Checklist 1 PRISMA-ScR checklist.

## Abbreviations

AUPRC: area under the precision–recall curve
DPA: deterioration prediction algorithm
JBI: Joanna Briggs Institute
ML: machine learning
PEWS: pediatric early warning system
PPV: positive predictive value
PRISMA-ScR: Preferred Reporting Items for Systematic Reviews and Meta-Analyses extension for Scoping Reviews
TRIPOD-AI: Transparent Reporting of a Multivariable Prediction Model for Individual Prognosis or Diagnosis With Artificial Intelligence
