# Supplementary material for: Technical Approaches to Predicting Acute Deterioration in Pediatric Inpatients: Protocol for a Scoping Review
Source: JMIR Res Protoc. 2026 Sep 24;15:e100097. doi: 10.2196/100097 (PMC13608229; doi:10.2196/100097)
Supplement: Supplementary file 2 [file resprot-v15-e100097-s001.docx]

# Search Strategies

## MEDLINE / Embase / HMIC (Ovid)

(exp pediatrics/ or  exp child/ or paediatric*.mp. or  pediatric*.mp. or  child*.mp. or adolescen*.mp. )

and

(exp early warning score/ or  exp "severity of illness index"/ or  acute illness severity.mp. or  early warning system.mp. or machine learning.mp. )

and

(deteriorat*.mp. or  escalat*.mp. or risk*.mp. or transfer*.mp. )

and

(prognos*.mp. or predict*.mp. or accuracy.mp. or performance.mp or valid*.mp. )

and

(inpatient.mp. or  hospital*.mp. or  ward*.mp. )

Filtered to 2000-2025

## Scopus

TITLE-ABS-KEY(paediatric* OR pediatric* OR child* OR adolescen*)

AND

TITLE-ABS-KEY("early warning score" OR "early warning system" OR "severity of illness" OR "acute illness severity" OR "machine learning")

AND

TITLE-ABS-KEY(deteriorat* OR escalat* OR risk* OR transfer*)

AND

TITLE-ABS-KEY(prognos* OR predict* OR accuracy OR performance OR valid*)

AND

TITLE-ABS-KEY(inpatient OR hospital* OR ward*)

Filtered to 2000-2025

## Web of Science

paediatric* OR pediatric* OR child* OR adolescen* (Topic)

AND

"early warning score*" OR "early warning system*" OR "severity of illness" OR "severity of illness index" OR "acute illness severity" OR "machine learning" (Topic)

AND

deteriorat* OR escalat* OR risk* OR transfer* (Topic)

AND

prognos* OR predict* OR accuracy OR performance OR valid* (Topic)

AND

inpatient* OR hospital* OR ward* (Topic)

AND

2000-2025 (Year Published)

## ACM DL

(Title:(paediatric* OR pediatric* OR child* OR adolescen*) OR Abstract:(paediatric* OR pediatric* OR child* OR adolescen*) OR Keyword:(paediatric* OR pediatric* OR child* OR adolescen*))

AND

(Title:("early warning score" OR "early warning system" OR "severity of illness" OR "acute illness severity" OR "machine learning") OR Abstract:("early warning score" OR "early warning system" OR "severity of illness" OR "acute illness severity" OR "machine learning") OR Keyword:("early warning score" OR "early warning system" OR "severity of illness" OR "acute illness severity" OR "machine learning"))

AND

(Title:(deteriorat* OR escalat* OR risk* OR transfer*) OR Abstract:(deteriorat* OR escalat* OR risk* OR transfer*) OR Keyword:(deteriorat* OR escalat* OR risk* OR transfer*))

AND

(Title:(prognos* OR predict* OR accuracy OR performance OR valid*) OR Abstract:(prognos* OR predict* OR accuracy OR performance OR valid*) OR Keyword:(prognos* OR predict* OR accuracy OR performance OR valid*))

AND

(Title:(inpatient OR hospital* OR ward*) OR Abstract:(inpatient OR hospital* OR ward*) OR Keyword:(inpatient OR hospital* OR ward*))

## Cochrane

#1

(paediatric* OR pediatric* OR child* OR adolescen*):ti,ab,kw

#2

((early NEXT warning NEXT score*)

  OR (early NEXT warning NEXT system*)

  OR (severity NEXT illness)

  OR (acute NEXT illness NEXT severity)

  OR (severity NEXT illness NEXT index)

  OR (machine NEXT learning)):ti,ab,kw

#3

(deteriorat*

OR escalat*

OR risk*

OR transfer*):ti,ab,kw

#4

(prognos*

OR predict*

OR accuracy

OR performance

OR valid*):ti,ab,kw

#5

(inpatient*

OR hospital*

OR ward*):ti,ab,kw

#6

#1 AND #2 AND #3 AND #4 AND #5
